# Parsing eye-tracking data of variable quality to provide accurate fixation duration estimates in infants and adults

**DOI:** 10.3758/s13428-012-0245-6

**Published:** 2012-09-06

**Authors:** S. V. Wass, T. J. Smith, M. H. Johnson

**Affiliations:** Centre for Brain and Cognitive Development, Birkbeck College, University of London, London, WC1E 7HX UK

**Keywords:** Fixation duration, Infant, Eyetracker methodology, Attention, Naturalistic, Free viewing

## Abstract

Researchers studying infants’ spontaneous allocation of attention have traditionally relied on hand-coding infants’ direction of gaze from videos; these techniques have low temporal and spatial resolution and are labor intensive. Eye-tracking technology potentially allows for much more precise measurement of how attention is allocated at the subsecond scale, but a number of technical and methodological issues have given rise to caution about the quality and reliability of high temporal resolution data obtained from infants. We present analyses suggesting that when standard dispersal-based fixation detection algorithms are used to parse eye-tracking data obtained from infants, the results appear to be heavily influenced by interindividual variations in data quality. We discuss the causes of these artifacts, including fragmentary fixations arising from flickery or unreliable contact with the eyetracker and variable degrees of imprecision in reported position of gaze. We also present new algorithms designed to cope with these problems by including a number of new post hoc verification checks to identify and eliminate fixations that may be artifactual. We assess the results of our algorithms by testing their reliability using a variety of methods and on several data sets. We contend that, with appropriate data analysis methods, fixation duration can be a reliable and stable measure in infants. We conclude by discussing ways in which studying fixation durations during unconstrained orienting may offer insights into the relationship between attention and learning in naturalistic settings.

## Introduction

Researchers studying infant behavior have traditionally used methods such as habituation/dishabituation to make inferences about infant cognition (Fantz, 1964). These experiments assess infants’ gross orienting behavior in controlled experimental paradigms by presenting the same stimulus repeatedly across a number of trials and measuring the durations of infants’ looks toward that stimulus on a time scale of seconds. These methods are useful as a way of assessing whether infants perceive that a stimulus has changed (for example, whether they can discern the difference between seven colored circles and eight); measuring the rate of change of looks over time is also useful as an index of infants’ speed of learning (Colombo & Mitchell, 2009). However, the restrictions of the paradigm means that its utility as an assessment of infants’ attention deployment in spontaneous, unconstrained settings may be limited (see Aslin, 2007). The basic experimental paradigm, in which a stimulus is presented repeatedly across a number of separate but immediately contiguous trials, is one that is seldom if ever encountered in the real world.

Researchers wishing to study infants’ spontaneous attention deployment in real-world settings have traditionally done this by video taping infants playing in naturalistic contexts and by hand-coding their direction of gaze post hoc (e.g., Choudhury & Gorman, 2000; Kannass & Oakes, 2008; Swettenham et al., 1998). These techniques have also yielded a number of vital insights into infant cognition—in particular, toward infants’ spontaneous orienting and learning behavior in social settings (e.g., Carpenter, Nagell, & Tomasello, 1998; Mundy & Newell, 2007). However, they also have limitations. Hand-coding infants’ direction of gaze from a video has a relatively low spatial resolution, and temporal resolution is also low: Although resolutions as high as 50 Hz can be obtained using video coding (Elsabbagh et al., 2009), this coding is extremely time consuming. It can take up to 5 h for one researcher to code 10 min of video, which limits the amount of data that can be processed using these methods. A more typical temporal resolution for video coding is 1 Hz (Kannass & Oakes, 2008; Ruff & Capozzoli, 2003; Wass, 2011; Wass, Porayska-Pomsta, & Johnson, 2011), although resolutions as low as 0.2 Hz (i.e., one sample every 5 s) are also sometimes reported. Because it is performed by humans, video coding is also more error prone.

Given the limitations of traditional methods, the advent of eyetrackers has brought a number of changes to the study of infant cognition (Aslin, 2012; Gredebäck, Johnson, & van Hofsten, 2010; Morgante, Zolfaghari, & Johnson, 2012; Oakes, 2011). As a noninvasive technique, eye tracking offers the potential to study infants’ spontaneous attention deployment in unconstrained, naturalistic settings. Relative to video coding, the advantage offered by eyetrackers is that the spatial resolution is much higher (typically ~1˚ of visual angle), as is the temporal resolution (typically, 50–500 Hz). Furthermore, the data processing can be performed automatically, meaning that there is effectively no limit on the volume of data that can be processed. This increased temporal and spatial resolution offered by eyetracker data opens up the possibility of analyzing in detail the subsecond correlates of attentional allocation—namely, how attention is apportioned through individual fixations and saccades.

When attending to a visual array, such as a natural visual scene or a sparse screen-based display, we spontaneously manifest a sequence of eye movements in order to ensure that light from objects of interest is projected onto the most sensitive part of the retina, the fovea (Holmqvist et al., 2011; Land & Tatler, 2009). When our eyes are stable (during a fixation), visual processing and encoding in working memory occurs. Fixations are separated by rapid, ballistic eye movements (saccades), during which visual sensitivity is suppressed to avoid the perception of blur as the image rapidly sweeps across the retina (Matin, 1974).

Within the adult literature, research has suggested that bottom-up visual features of scenes such as edges and motion (Itti & Koch, 2001), luminance (Loftus, 1985), or blur (Mannan, Ruddock, & Wooding, 1995) can influence fixation duration, as well as top-down factors such as viewing task and personal preference (Henderson, Weeks, & Hollingworth, 1999; Yarbus, 1967; see also Nuthmann, Smith, Engbert, & Henderson, 2010; Tatler & Vincent, 2008). Research has also suggested the existence of an internal stochastic timer mechanism that triggers saccades irrespective of immediate processing (Engbert, Longtin, & Kliegl, 2002; Henderson & Smith, 2009).

Relatively less research has examined fixation durations during spontaneous orienting in infants. When infants of 1–2 months examine static visual stimuli, they tend to view each stimulus in a series of long fixations that are located close together (Bronson, 1990). By 3–4 months, however, they show a more controlled, strategic method for scanning static stimuli, with a greater proportion of shorter (<500 ms) fixations (Bronson, 1994; see also Hunnius Geuze, & van Geert, 2006). Bronson (1994) also reported that fixation durations in 6-week-old infants are relatively more influenced by whether the fixation falls on a stimulus contour. The change in orienting style is thought to be mediated by a reduction in the early difficulties that infants encounter with disengaging their attention—known as “sticky fixation” or “obligatory attention” (Hood & Atkinson, 1993; see also Atkinson, 2000; Hunnius, 2007; Johnson, 1993, 2010; Johnson, Posner, & Rothbart, 1991). By 4 months, however, problems with disengaging from static stimuli have largely dissipated, although the problem of “sticky fixation” may be more long-lasting with dynamic stimuli (Bronson, 1994; Hood & Atkinson, 1993). Bronson (1990) examined changes in infants’ scanning to geometric patterns across the 2- to 14-week period. He found that as infants grew older, they became increasingly disposed to scan between different stimulus features while viewing static stimuli. When the stimulus was moving, however, the infants’ scanning characteristics reverted to those typically found at younger ages, suggesting that “sticky fixation” behaviors may persist longer into development for dynamic than for static stimuli.

A substantial body of research with adults has pointed to the validity and reliability of fixation durations as an index of online cognitive processing (see, e.g., Nuthmann et al., 2010; Rayner, 1998). Within the infant literature, however, a number of important research questions remain unaddressed. The degree to which fixation durations are influenced by endogenous versus exogenous factors in infancy, the degree to which differences in fixation duration relate to individual differences on other cognitive measures, and the degree to which fixation durations can be a marker for early disrupted development are all questions that remain to be explored.

### Analyzing fixation duration – preexisting fixation-parsing algorithms

Most of the work described above with infants has used hand-coding techniques to analyze fixation durations. Bronson (1990, 1994) recorded infants’ gaze positions using an early corneal reflection-based device, replayed infants’ recorded position of gaze (POG) onto a rear projection screen post hoc, and identified fixations by hand. De Barbaro, Chiba, and Deak (2011) took close-up video footage of an infant’s eye and defined fixations as instances in which the eye remained static for at least 230 ms (seven frames at 30 fps). Although it can be done in a variety of ways, any type of hand-coding is extremely labor intensive, and both temporal and spatial resolution are lower than that offered by eyetrackers, suggesting the desirability of finding an automated solution.

Methods for recording eye movements have been around for over a hundred years (Wade & Tatler, 2005), but only with the relatively recent advent of high-speed infrared (IR) cameras and fast computers has eye tracking become noninvasive enough to be used with infants. Most remote, video-based eyetrackers operate by illuminating the user with IR light and using computer vision techniques to identify either a dark pupil (created by off-camera-axis illumination) or a bright pupil ("red eye effect"; caused by on-camera-axis illumination) (for a more detailed summary, see Holmqvist et al., 2011). The IR illuminators also create bright glints off the user's cornea. By triangulating the movement of the pupil center relative to these glints when the user is looking at five to nine calibration points on a screen, the eye-tracking software is able to build a 2-D model of the user's eye movements relative to the screen. Once the eye model has been built, the tracker can identify the location of a user's gaze on the screen in real time. Eyetrackers vary in their sampling speed and spatial accuracy, in whether they are binocular or monocular, in whether they require the user's head to be stabilized or allow head movement within a tracking volume, and in the complexity of the eye model (2-D or 3-D), but the general principles of IR pupil and corneal reflection tracking are similar across systems (Holmqvist et al., 2011).

The raw gaze data returned by an eyetracker include periods during which the eyes are relatively stable and visual encoding occurs (fixations), periods when the velocity of the gaze is high (saccadic eye movements), periods during which moving objects are tracked relative to the viewer (smooth pursuits), periods during which the viewer moves in depth (vergence), and periods when gaze is lost due to blinks. There are three standard measures used to separate fixations from other eye movement events: dispersal, velocity, and acceleration (for more detailed reviews, see Duchowski, 2007; Nyström & Holmqvist. 2010; Wade, Tatler, & Heller, 2003). Dispersal is defined as the distance (expressed as pixels or degrees of visual angle) in the POG reported by the eyetracker between samples; velocity and acceleration are differentials derived from the POG.

It is traditional to draw a distinction between dispersal- and velocity-based algorithms (e.g., Blignaut, 2009; Holmqvist et al., 2011; Nyström & Holmqvist, 2010; Shic, Chawarska, & Scassellati, 2008, 2009; van der Lans, Wedel, & Pieters, 2011). Dispersal-based algorithms search for periods in which the reported POG remains below a displacement threshold that is often user-defined. Thus, for example, they identify a period of raw gaze data as belonging to a fixation by starting with a window size of the minimum fixation duration (e.g., 80 ms) and expanding it until the average displacement of the eyes during the window is greater than the displacement threshold (e.g., 0.5°). Periods not identified as fixations using this method are assumed to be either saccades or periods of lost data. Velocity-based algorithms, in contrast, search for saccades (i.e., instances in which the rate of change of POG surpasses a threshold) and labels periods between saccades as fixations (Blignaut, 2009; Nyström & Holmqvist, 2010). Velocity-based algorithms can either use one fixed velocity criterion (e.g., 30°/s; SR Research; Eyelink User Manual, 2005) or have a variable velocity threshold set according to the level of noise in the data (e.g., Behrens et al., 2010). The “ramping up” of the saccade velocity can also be identified via an acceleration threshold (e.g., 8,000°/s^2^; SR Research). Versions of all three algorithms are in widespread use in several commercial software packages provided by companies such as Applied Science Laboratories, SensoMotoric Instruments, Tobii Technology, and SR Research.

One issue that has received attention in the literature is that the fixations returned by traditional velocity- and dispersal-based algorithms appear highly sensitive to user-defined parameter settings, such as the level at which the dispersal or velocity threshold is set. Karsh and Breitenbach (1983) showed that varying the parameters of a fixation detection algorithm led to qualitative differences in the scan patterns that emerged (see also Widdel, 1984). Shic et al. (2008) showed that changing the user-definable parameters of a distance-dispersal algorithm can lead to different patterns of between-group or between-individual differences in mean fixation duration. Thus, for example, they found that when the dispersal setting of their fixation duration algorithm was set at below 3° (equivalent to a radius of 1.5°), mean fixation duration when viewing faces was greater than that for viewing color blocks, but that when the dispersal setting was set to above 3°, mean fixation duration for blocks was returned as being greater than that for faces. Shic et al. (2009) similarly reported that changing the dispersal levels could reverse the pattern of typically developing versus autism spectrum disorder group differences on fixation duration. It should, though, be remembered that 3° (corresponding to 125 pixels in a 1,024 × 768 pixel monitor at 60-cm viewing distance) is 20% higher than the maximum recommendation for dispersal fixation algorithms in adults (Blignaut, 2009).

### Analyzing fixation duration – the special challenges posed by low-quality infant data

Gathering accurate eye movement recording from infants is significantly more difficult than with adults for a variety of reasons. Adults are complicit during eye-tracking recording. They can be persuaded to keep their head on a chinrest and to minimize blinks or head movements and can be expected to behave in line with the demands of the task. By comparison, infants and children below the age of about 4 are likely to be less compliant than adult viewers and are more likely to move during eye tracking. To compensate for this, head movement eyetrackers intended for use with infants allow for movement within a “head-box.” For example, the Tobii 1750 has a head-box 30 × 16 × 20 cm in diameter centered 60 cm from the screen (Tobii Eye Tracker User Manual, 2006). Analysis of the accuracy of the gaze data reveals that movement of the head toward the edges of this head-box, as well as changes in luminance caused by room lighting or the angle of the user's head in relation to light sources, can all significantly decrease accuracy (Tobii test specification, 2011). Additionally, the recording may also include periods during which gaze data are absent completely. This is due either to the head moving out of the head-box or to either the corneal reflection or the pupil image becoming unidentifiable for some other reason.

**Fig. 1:**
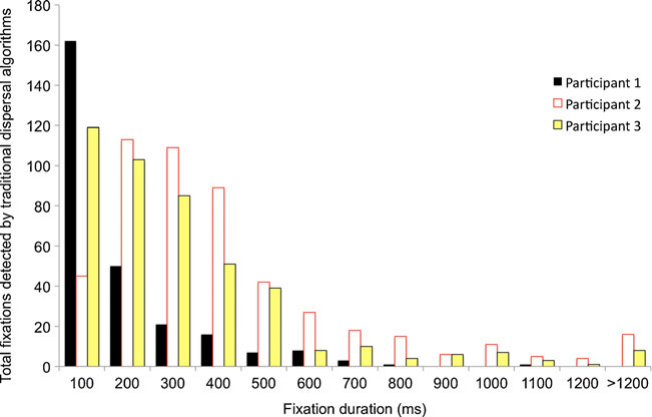
Frequency distribution showing fixation durations returned by a standard dispersal algorithm from 3 sample infant participants

Figure 1 shows the results when a standard dispersal-based algorithm designed for processing adult data is applied to infant data. This algorithm is the fixation detection algorithm supplied with Clearview 2.7 (Tobii Eye Tracker User Manual, 2006) at the default settings (dispersal threshold of 30 pixels [corresponding to 0.9°] and a minimum temporal duration of 100 ms). As with all stimuli presented here, the viewing material was presented on a monitor at a 60-cm viewing distance subtending 24° × 29°.

The figure shows frequency distributions of fixation durations returned for 3 sample participants. All 3 participants were typically developing 6-month-old infants viewing a 200 s corpus of dynamic viewing material. For 1 participant (participant 2), a positively skewed normal distribution with a mode of 279 ms, a mean of 516 ms, and a median of 379 ms was returned. The shape of this distribution is broadly similar to the distributions of fixation durations described in the adult literature (e.g., Henderson, Chanceaux, & Smith, 2009; Nuthmann et al., 2010; Nyström & Holmqvist, 2010; Tatler & Vincent, 2008). For the other participants, however, a radically different distribution was returned. Participant 1, for example, shows an inverse exponential distribution with a mode of 100 ms, a mean of 217 ms, and a median of 160 ms; more than three times as many fixations are being identified in the 100- to 200 ms range than in the 200- to 300-ms range. Participant 3 is intermediate, with a mean of 354 ms, a mode of 100 ms, and a median of 279 ms. For 2 of the 3 participants, the mode is 100 ms, which is the shortest possible fixation duration (due to the minimum temporal duration criterion identified above, all fixations shorter than this are excluded). Furthermore, there are extremely large differences in the mean fixation durations being reported across the 3 participants (from 217 to 516 ms).

Although it is possible that such radically differing response distributions arise because of differences in infants’ spontaneous orienting behavior, we wished to assess the possibility that it may be artifactual in origin. In particular, we considered the possibility that infant eyetracker data may differ in some way from adult data, so that a fixation detection algorithm designed for adult data may be suboptimal for infant data. We were able to find no discussion of this issue in the literature.

**Fig. 2:**
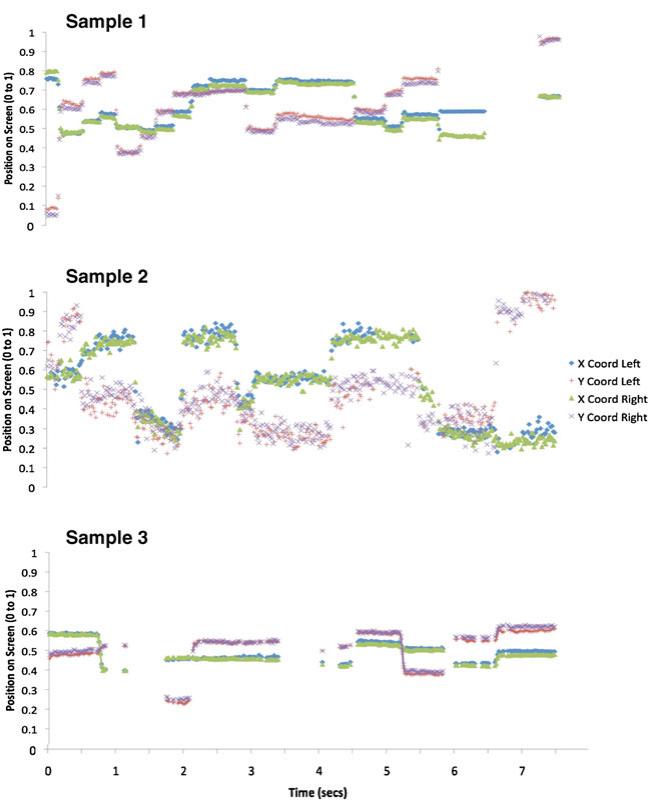
Sample data plots. Data were taken from 3 infant participants. Sample 1 shows high-quality tracking, sample 2 shows low precision, and sample 3 shows flicker. The exact way in which these terms are used are defined in detail in the text

In order to assess how data quality might differ between adult and infant eyetracker data, we first plotted samples of raw data. Figure 2 shows examples of data quality as 3 participants (typically developing 11-month-old infants) viewed an identical 8 s dynamic clip of multiple actors talking concurrently against a busy background. These data were recorded using a Tobii 1750, with stimuli presented in MATLAB using the Talk2Tobii toolbox.

Visual inspection of these data suggest considerable interindividual variations (also known as idiosyncrasies) in data quality, and also that there may be more than one separable dimension of data quality. The data from participant 1 appear to be of high quality relative to adult data (see e.g. Holmqvist et al., 2011). It is unbroken (i.e. continuous); sections where the eye is stationary (fixations) are clearly distinguishable from sections where the eye is transiting (saccades). Participants 2 and 3, however, show lower quality data. Participant 2 shows greater variance in reported POG between one sample and the next. We assume that this individual's eye is stable during fixation, because from the infant oculomotor literature, we were able to find no reports of such high-frequency (50 Hz) “jitter” in infant eye movement behavior (Atkinson, 2000; Bronson, 1990, 1994; Johnson, 2010; see also Holmqvist et al., 2011), and our own video analysis of infant eye movements during viewing confirmed this conclusion. We concluded, therefore, that this high-frequency variance arises from lower than normal precision in the reporting of the POG from the eyetracker —that is, a larger than normal random error arising from one iteration to the next between the participant’s actual POG and the POG as reported by the eyetracker.

Participant 3 shows a different problem. For this individual, the precision appears to be as high as that for participant 1. However, contact with the eyetracker appears to be “flickery” —that is, absent for periods of time of variable length. Because head movement is unconstrained, all infant eyetrackers may have increased problems of unreliability—that is, instances in which the infant is looking to the screen, but either the pupillary reflection or glint is unavailable or judged unreliable, leading to no POG being recorded. Visual inspection of the raw data obtained from infants suggested a high degree of variability in these periods of data loss. Contact is lost for variable periods of time ranging from a single iteration (20 ms) through to longer periods. In other examples than that shown here, contact may also occasionally be lost for one eye but not for the other.

In order to quantify these different aspects of data quality, we analyzed a corpus of 300 s of dynamic viewing material presented to 17 six-month-old infants, 16 twelve-month-old infants, and 16 adult viewers. Stimuli were presented on a Tobii 1750 eyetracker using ClearView 2.7. The viewing material presented was a collection of low-load dynamic clips of objects moving against a blank background (described in more detail in Dekker, Smith, Mital, & Karmiloff-Smith, 2012).

#### Flickery or unreliable contact with the eyetracker

We quantified this aspect of data quality in two ways. First, we reported on the total proportion of unavailable data across the whole trial. This was calculated as the proportion of data obtained as a function of the total amount of viewing material presented. Second, we calculated a different measure of flickery contact, which is the mean duration (in milliseconds) of each raw data segment. These two measures allow us to differentiate between (1) cases in which the participant showed unbroken looking data during the first half of a trial, followed by completely absent data for the second half of the trial, and (2) instances in which the infant was looking continuously throughout the trial but contact with the eyetracker was inconsistent throughout (as shown in sample 2; see Fig. 2).

#### Precision: Variance in reported position of gaze

Quantifying this aspect of data quality is challenging because simply calculating the between-sample variance (i.e. the average interiteration variability) leaves open the possibility that interindividual differences occur simply because one individual saccades more around the screen than does another (see the related discussion in Holmqvist et al., 2011, chap. 11). In order to quantify unreliability in reported POG, therefore, we performed the following calculation. First, we performed an initial coarse dispersal-based parsing to eliminate all saccades. (Given that most of the issues we have identified concern the false positive identification of saccades and that, therefore, most of the data segments identified by the dispersal-based filtering are still real fixations, albeit incomplete ones, we felt that this method was free of any systematic bias.) For each of the data segments remaining, we then calculated the average variance (i.e., the average Euclidean distance of each individual sample within each fixation from the central point of that fixation). This is expressed in degrees of visual angle. High variance indicates low precision—that is, inaccurate or inconsistent reporting in POG.

Univariate ANOVAs were conducted on these results that suggested that all three parameters vary significantly as a function of age. Proportion of unavailable data is higher in 6-month-olds (*M* = .33 [*SD =* .17]) and 12-month-olds (.31 [.16]) than in adults (.06 [.06]) and varies as a function of age, *F*(1, 46) = 19.52, *p* < .001. Mean duration of raw data fragments is lower in 6-month-olds (*M* = 2.3 s [*SD* = 1.8]) than in 12-month-olds (4.3 [3.5]) and adults (9.9 [9.7]) and varies significantly as a function of age, *F*(1, 46) = 6.68, *p* = .01. Variance in reported POG follows the opposite pattern and is lower in 6-month-olds (*M* = 0.18° [*SD* = 0.05°]) and 12-month-olds (0.18° [0.02°]) than in adults (0.25° [0.05°]) and varies as a function of age, *F*(1, 46) = 12.3, *p* < .001.

Bivariate correlations were also calculated to examine whether these different parameters of data quality intercorrelate with each other. Although proportion of unavailable data and flicker (i.e., mean duration of raw data fragments) correlated in each of the three separate samples we looked at [6 months, *r*(1, 16) = −.62, *p*(two-tailed) = .01; 12 months, *r*(1, 15) = −.59, *p*(two-tailed) = .02; adults, *r*(1, 15) = −.53, *p*(two-tailed) = .03], we found no consistent pattern of correlations between flicker and precision (i.e. variance in reported POG) [6 months, *r*(1, 17) = .11, *p*(two-tailed) = .68; 12 months, *r*(1, 16) = −.09, *p*(two-tailed) = .75; adults, *r*(1, 16) = −.39, *p*(two-tailed) = .15]. This suggests that flicker and precision are independent dimensions of data quality.

#### Evaluating how data quality relates to fixation duration in an infant data sample

Using the same sample, we then evaluated whether relationships could be identified between data quality and the fixation durations returned by standard dispersal-based algorithms. Fixation parsing was performed by the fixation detection algorithms supplied with Clearview 2.7 (Tobii Eye Tracker User Manual, 2006) at the default settings as described above. However, to the best of our knowledge, the points we include in this discussion should apply equally to all of the preexisting fixation detection algorithms discussed in the introductory section.

**Fig. 3:**
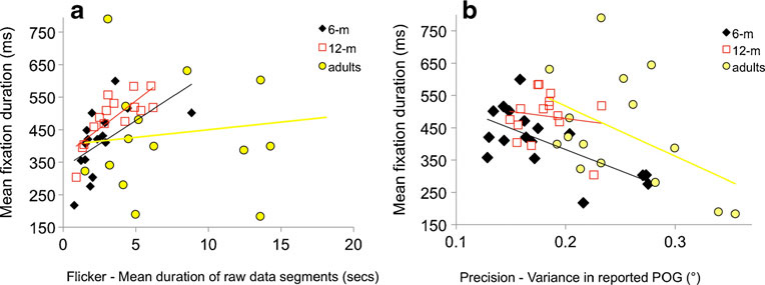
Quantifying how fixation duration varies as a function of data quality. The relationship between fixation durations (as analyzed using standard dispersal algorithms) and data quality is evaluated in two ways: **a** flicker, the mean duration of raw data segments, And **b** precision, the variance in reported point of gaze (POG)

Figure 3a shows the relationship between fixation duration as measured using standard dispersal algorithms and mean duration of raw data segments. Across the three different age groups we examined consistently positive correlations were found, suggesting that longer data segments (i.e. less flickery data) were associated with longer fixation durations as assessed using standard dispersal algorithms. Nonparametric bivariate correlations analyses suggested that the observed relationships were significant for the 6-month group, (r(1, 17) = .66, p = .004), marginally non-significant for the 12-month group (r(1, 16) = .47, p = .07) and not significant for the adult group r(1, 15) = .19, p =.50). Figure 3b shows the relationship between fixation duration as measured using standard dispersal algorithms and variance in reported POG. Here, bivariate correlations suggested that the relationship was significant for the 6-month-old group, *r*(1, 16) = −.673, *p*(two-tailed) = .003, but not for the 12-month-old group, *r*(1, 14) = −.141, *p*(two-tailed) = .626, or the adult group, *r*(1, 15) = −.469, *p*(two-tailed) = .078. These findings represent significant methodological confounds that substantially limit the interpretability of the results of standard dispersal-based fixation detection paradigms.

#### Comparing with hand-coded data

In order further to understand the relationship between data quality and performance of the standard dispersal-based algorithm, we compared the performance of the standard dispersal-based algorithm with the results of hand-identified fixations. Hand-coding of gaze data is sometimes performed in order to identify a “gold standard” for fixation detection—that is, a “true” parsing of gaze data into fixations and saccades with which the results of the automated processing can then be compared (Holmqvist et al., 2011; Munn, Stefano, & Pelz, 2008; Tatler, Gilchrist, & Land, 2005).

In order to do this we trained a novice coder (who was not one of the authors on the article and was naive as to expected outcomes) to identify fixations by hand, on the basis of a visual output of the raw gaze data returned by the eyetracker. The coder viewed the data in 8 s segments containing plots of the *x-*, *y*-coordinates and the velocity, with time on the *x*-axis. The coder was asked to identify fixations as segments in which the POG stayed static (i.e., deviated by <0.5º) for longer than 100 ms. The coder was instructed to ignore fixations in which contact was lost either during the fixation or during the saccades before and after. The coder was also instructed to record only fixations in which the saccades that marked the start and end of the fixation were both genuine (i.e. in which the saccade (period of high velocity) was clearly distinguishable from the fixations (periods of low velocity) before and after). These were distinguished from “false saccades” (in which the period of high velocity movement was not clearly distinguishable from the periods of low velocity movement before and after). Sections of data that showed these “false saccades” were excluded from the analysis. The start times and end times of fixations that were considered valid were recorded by the coder to the nearest 20 ms.

This hand-coding was conducted on a sample of data from 5 typically developing 11-month-old infants watching 150 s of mixed static and dynamic viewing material consisting of pictures and video clips of faces and objects.

**Fig. 4:**
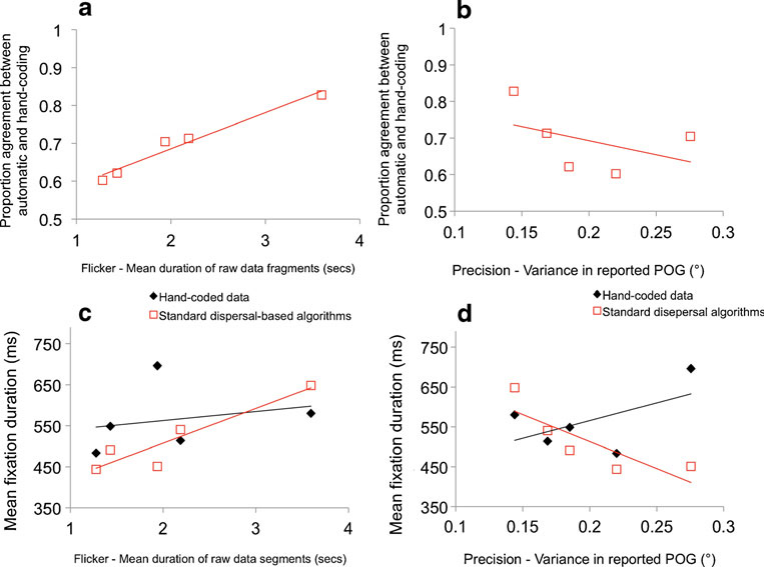
Quantifying performance of a standard dispersal-based algorithm relative to hand-coding as a function of data quality. **a** Relationship between flicker and proportion agreement between automatic and hand-coding. **b** Relationship between precision and proportion agreement between automatic and hand-coding. **c** Relationship between flicker and mean fixation duration, as processed using both hand-coding and standard dispersal algorithms. **d** Relationship between precision and mean fixation duration, as processed using both hand-coding and standard dispersal algorithms

Figures 4a and b show reliability—that is, the proportion of agreement between the results of the automatic coding and hand-coding. Figure 4a shows a strong relationship between degree of flicker in an individual’s data and the proportion of agreement between automatic and hand-coding. For the individual with less flickery (i.e., high-quality) data (mean raw data fragment duration, 3.8 s), we found interrater agreement of .83 (corresponding to Cohen’s κ of 0.66), whereas lower quality (i.e., more flickery) data shows an interrater agreement of .60 (Cohen’s κ = 0.20). The observed relationship between raw data fragment duration and the reliability of the standard dispersal algorithm was very strong, *r*(1, 4) = .98, *p* = .003. Figure 4b shows that for high-precision data (i.e., low variance in the reported POG) agreement is high between hand- and automatic coding, but for low-precision data, the reliability of the algorithm is poorer. This relationship is weaker than that between flickeriness and reliability, *r*(1, 4) = −.70, *p* = .19.

The relationships documented above leave open, however, the question of whether for lower quality data, standard dispersal-based algorithms tend to under- or overestimate fixation durations, relative to hand-coding. Figure 4c shows a comparison between mean duration of raw data segments and fixation duration as parsed using standard dispersal-based algorithms. Although the results must be interpreted with caution due to the small sample size, the results of this figure are consistent with the relationship suggested by Fig. 3. For individuals with low-quality flickery data (i.e., short duration of raw data segments), the standard dispersal-based algorithm consistently underestimates fixation duration, relative to the hand-coding, whereas for individuals with higher-quality data (i.e., long raw data segments), the algorithm consistently overestimates fixation duration. There is thus a significant correlation between flickeriness and fixation duration for the results of standard dispersal-based fixation parsing, *r*(1, 4) = .90, *p* = .04 (more flickery data associated with shorter fixation duration) but no correlation between flickeriness and fixation duration for the hand-coding. Figure 4d shows a comparison between precision and fixation duration. A similar interaction appears to be present: For high-precision (i.e., low-variance) data, the dispersal-based parsing algorithm tends to overestimate fixation duration relative to the hand-coding, whereas for low-precision (i.e., high-variance) data, the dispersal-based parsing algorithms tend to underestimate fixation durations. Again, we identified a significant relationship between precision and fixation duration as parsed using the standard dispersal-based algorithm, *r*(1, 4) = −.90, *p* = .04 (lower precision data associated with shorter fixation duration), but no correlation between precision and fixation duration for the hand-coding.

#### A simulation to assess how data quality might affect performance on a standard dispersal algorithm

The analyses above suggest that when parsing is performed using standard dispersal-based algorithms, individuals for whom the raw data was more flickery tend to be returned as showing shorter fixation durations. They also suggest that individuals for whom the raw data showed lower precision in the reported POG are returned by the standard dispersal algorithm as showing shorter fixations.

What, though, are the mechanisms driving these observed relationships? In order better to understand this issue, we conducted a simulation in which a single sample of high-quality data (5 min of viewing data taken from a typically developing 11-month-old infant viewing a mixed dynamic/static viewing battery) was subjected to two simulations.

**Fig. 5:**
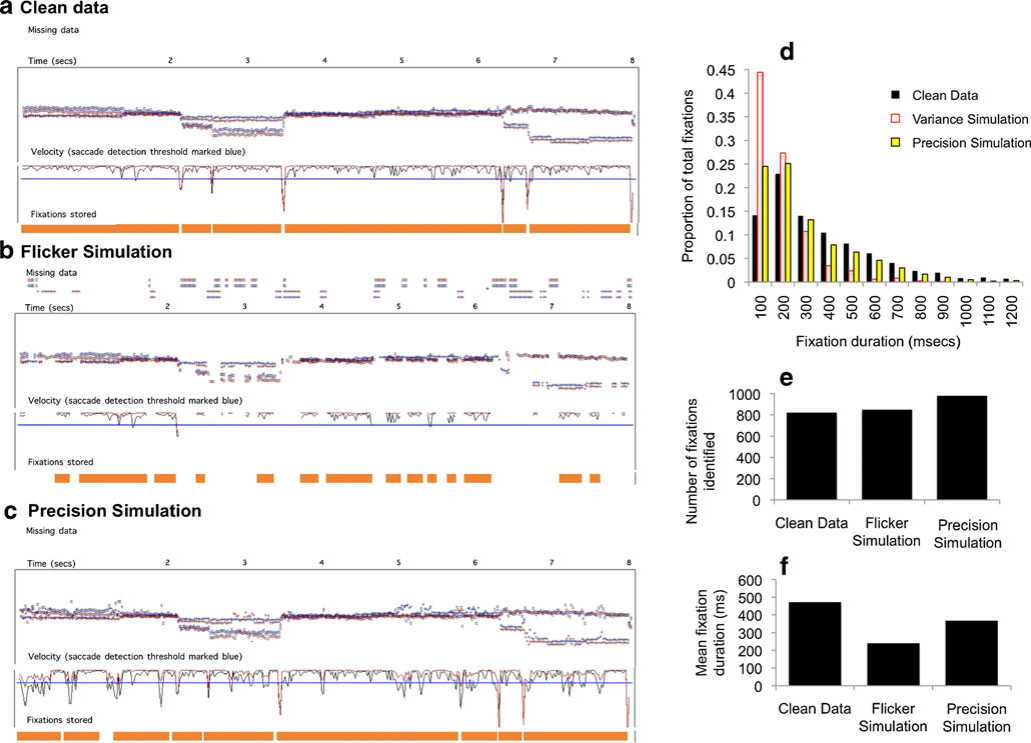
Evaluating the performance of a standard dispersal-based algorithm using simulations of the effect of flicker and precision. **a** Initial sample of clean data. The *x- *and *y*-coordinates are plotted, followed by a velocity plot (with the velocity threshold marked in blue). The fixations being returned by the standard dispersal algorithm are marked below the text as orange bars. **b** The same data distorted to simulate the effect of flickeriness. The orange bars show the fixations that are returned when these data are processed using standard dispersal algorithms. **c** The same data distorted to simulate the effect of low precision in reported point of gaze (POG). **d**) Frequency distribution showing the fixation durations returned in the three conditions. **e** Number of fixations identified in the three conditions. **f** Mean fixation duration returned by the algorithm in the three conditions

**Fig. 6:**
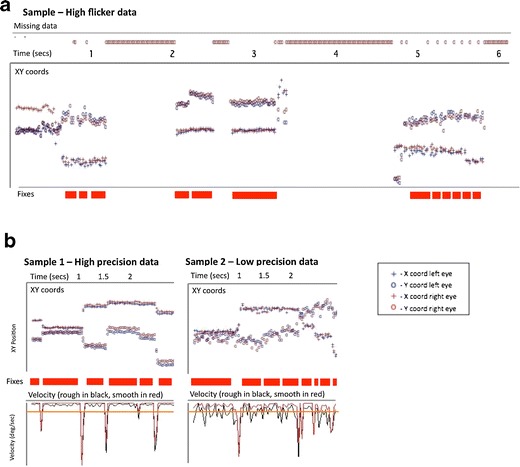
Sample plots of eyetracker data obtained from typically developing 11-month-old infants viewing static (still images) that illustrate the issues identified in Fig. 5. In each case, time (in seconds) has been plotted along the *x*-axis. Vertically from top to bottom each data plot shows missing data, raw *x*- and *y*-coordinates, and fixations identified by a standard dispersal algorithm (drawn as red bars). **a** Instance of a participant showing high flicker in the contact with the eyetracker, with multiple incomplete fixations being stored. **b** Samples from 2 participants, one of whom shows high precision data (with accurate identification of fixations), the other of whom shows low precision data (with inaccurate identification of fixations). On these plots, the velocity derived from the *x*- and *y*-coordinates has been plotted, along with the velocity threshold (drawn as an orange line). In sample 2, it can be seen that the velocity threshold is being surpassed due to random sampling error between iterations, leading to the false identification of fixations

First, a flicker simulation was conducted to replicate the nondeterministic dropout observed in our data (see Figs. 5 and 6). This was implemented in MATLAB by reprocessing the data iteration by iteration; if data for the previous iteration had been present, the algorithm removed data with a 5% likelihood, and if data for the previous iteration had been absent, the algorithm removed data with a 25% likelihood. This process was performed independently for the two eyes. Second, a precision stimulation was conducted to replicate the problems of unreliable reporting in POG. Again, this was implemented to replicate the nondeterministic nature of the data corruption we found in our data, in which we often encountered brief “bursts” of noise. In our simulation, a burst of noise was triggered with a 2.5% likelihood, and once a burst was initiated, it was continued with a 20% likelihood. During a noise burst, Gaussian noise (±0.1 of screen proportion, corresponding to 2.4°) was added to the data. The effect of these simulations was then tested on a re-creation of a standard dispersal algorithm that we programmed (because the preexisting manufacturer-supplied fixation detection algorithms do not allow for the processing and reprocessing of the same data set). This algorithm was programmed exactly to follow the analysis protocol implemented in Clearview 2.7 (Tobii Eye Tracker User Manual, 2006).

Why does flickery or unreliable contact with the eyetracker pose a potential challenge to the accurate identification of fixations? Because most of the preexisting fixation detection algorithms treat an instance in which contact with the eyetracker was lost during a fixation as signaling the end of that fixation. Flickery data may, therefore, lead to the storing of multiple incomplete fixations—that is, fixations that are stored as multiple separate fixations, whereas in fact they are part of one long fixation. This is indeed the effect that we appear to observe in our flicker simulation (shown in Fig. 5b). The frequency distribution of fixations detected shows an increase in the proportion of short (<200 ms) fixations being identified (Fig. 5d). The effect on mean fixation duration of the flicker simulation is also substantial (Fig. 5f): Mean fixation duration is 471 ms for the clean data, 240 ms for the flicker simulation, and 367 ms for the low precision simulation. Figure 6a shows an example of data obtained from a typically developing 11-month-old infant in which a similar effect appears to have occurred.

Why is low precision a potential challenge in obtaining accurate estimations of fixation durations? Most of the commonly available fixation duration parsing algorithms operate either via a displacement threshold, according to which a fixation is treated as ending following a change in POG above a certain, often user-defined, displacement threshold (see Blignaut, 2009; Holmqvist et al., 2011), or via a velocity threshold, according to which a fixation is treated as ending following an increase in velocity above a certain velocity threshold. (If data are obtained at a constant rate, these two criteria are the same). Figure 5c shows the effect of the low-precision simulation on performance of the standard dispersal algorithm. The velocity plot shows that the noise bursts lead to an increase in the velocity (i.e., the rate of change of reported POG from one sample to the next) that exceeds the saccade detection threshold, leading to a saccade being incorrectly identified. Thus, multiple incomplete fixations are stored, instead of one long fixation. Figure 5e shows that the number of fixations being identified is higher in the low-precision simulation. Figure 5d shows that an increased number of very short fixations are also being stored in this simulation, which is associated with shorter mean fixation duration (Fig. 5f). This effect is less strong than in the flicker simulation, although it may have increased if the amplitude of the noise added to the data was increased. Figure 6b shows a sample of eyetracker data in which a similar effect has occurred.

#### Conclusions

These analyses indicate that when parsing is performed using standard dispersal-based algorithms, individuals for whom the raw data were more flickery (i.e., there was a greater number of instances in which the eyetracker was unable to detect where they were looking) tend to be returned as showing shorter fixation durations than when the data is analyzed using standard dispersal algorithms (see Figs. 3 and 4). They also show that individuals for whom the raw data showed lower precision (i.e., higher variance in the reported POG, presumably because of a larger than normal random error between the participant’s actual POG and the POG as reported by the eyetracker) are returned by the standard dispersal algorithm as showing shorter fixations (see Figs. 3 and 4). The fact that flicker and precision do not correlate with each other suggests that they are two independent dimensions of data quality that can separately influence the accuracy of fixation parsing.

The simulation we conducted has given insight into the mechanisms that may be driving these relationships (see Figs. 5 and 6). Flickery contact appears to influence fixation durations returned by standard dispersal algorithms because almost all fixation detection algorithms are set up to treat an instance in which contact is lost during a fixation as signaling the end of that fixation. Therefore, flickery contact is associated with multiple incomplete fixations being stored. Low-precision data appear to influence fixation duration as identified by standard dispersal algorithms because inconsistent reporting of the POG leads to bursts of high velocity, which can lead to the saccade detection threshold being fired erroneously. Both of these conclusions are supported by inspection of the raw and semiprocessed data sets (Fig. 6).

Both of these findings represent potentially serious and independent confounds that substantially limit the interpretability of fixation duration as assessed using standard dispersal-based algorithms. These findings have been based on a replication of the standard dispersal-based algorithm implemented in Clearview 2.7, but from our analysis of similar algorithms by other groups, we can find no reason why these same artifacts should not also affect the performance of other algorithms when processing low-quality data. These findings agree with those of other authors who have questioned the reliability of standard dispersal-based fixation detection algorithms (Blignaut, 2009; Camilli, Nacchia, Terenzi, & Di Nocera, 2008; Karsh & Breitenbach, 1983; Shic et al., 2008, 2009).

## Designing new fixation detection algorithms to cope with low-quality infant eyetracker data

We wanted to design fixation-parsing algorithms that avoid the problems documented above. The algorithms described in this section can be downloaded from http://www.cbcd.bbk.ac.uk/people/scientificstaff/sam. All of the specific thresholds we describe below have been implemented in these algorithms so that the user can adjust them as (s)he sees fit.

One method for dealing with the problem of low precision in reported POG would be to use a variable velocity threshold that changed contingent on the reliability of the reported POG on a participant-by-participant basis (cf. Behrens, MacKeben, & Schroeder-Preikschat, 2010; Nyström & Holmqvist, 2010; Tole & Young, 1981; van der Lans et al., 2011; discussed in Holmqvist et al., 2011). However, we decided not to do this in the present case because we were concerned that it would lead to a difference in the degree to which genuine small saccades were classified as sub- or suprathreshold, meaning that poorer quality data would tend to show longer fixations than higher quality data. Thus, for example, increasing the velocity threshold would lead to small saccades passing just subthreshold, and a variable velocity threshold would lead to saccades of this magnitude passing undetected for some participants but not for others (Blignaut, 2009; Camilli et al., 2008). Therefore, instead, we decided to implement a novel approach: to use a single velocity threshold but, additionally, to incorporate a number of criteria for detecting whether or not the fixations being detected were genuine. Thus, our velocity threshold was set for all participants at the same level, but additionally, a number of post hoc criteria were devised so that incorrectly identified fixations were filtered out.

**Fig. 7:**
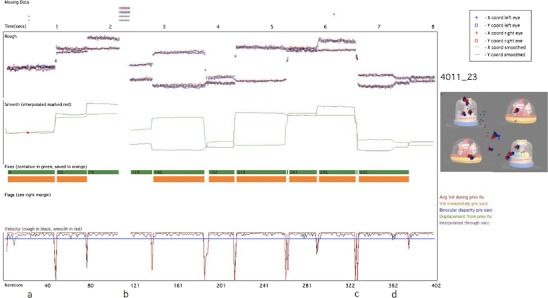
Sample processed data plot. The time (in seconds) is plotted along the *x*-axis, top of the screen, and the number of iterations along the *x*-axis, bottom of the screen. Vertically from top to bottom, we have plotted the following: missing data (above the time axis); rough (i.e. raw) data, with separate *x*- and *y*-coordinates for left and right eyes; smoothed data (with interpolated sections marked in red); fixations (tentative fixations marked as a thick green line, and fixations that have passed all of our verification checks marked in orange); bad data flags (absent in this sample); and velocity (calculated from raw data in black and from smoothed data in red). The velocity threshold of 35ºs-1 is drawn as a thin blue line. On the right hand side of the screen, the participant and trial numbers are drawn (in this case ‘4011_23’), along with the stimulus the infant was viewing, with their gaze positions (left eye, blue; right eye, red) drawn on the screen. The letters a–d below the bottom axis refer to examples we describe in the text

The algorithms work post hoc in a feedforward manner. The chain of processing is as follows: (1) smoothing; (2) interpolating; (3) velocity thresholding; (4) rejection of false fixations and saccades. These steps are described in detail below. In order to illustrate the discussion that follows, we have also included in Figs. 6 and 7 two sample data plots of 8 s sections of data, to illustrate the different types of data that our algorithms were designed to cope with.

### Smoothing

**Fig. 8:**
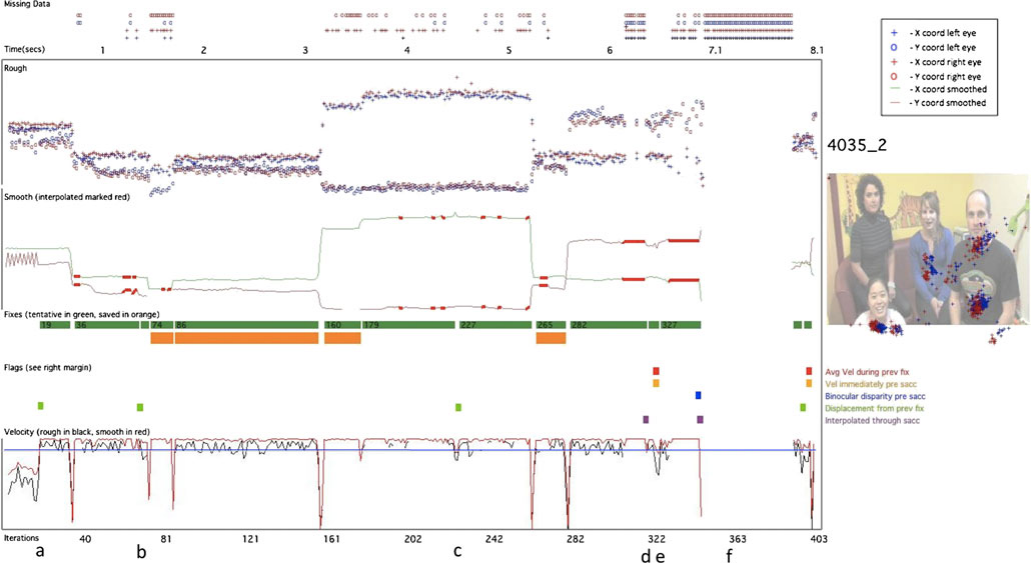
Same layout as that in Fig. 7. This is a sample of lower quality than that shown in Fig. 7. Instances in which our false fixation and saccade detection flags have fired are drawn as colored squares above the velocity plot. Tentatively identified fixations are marked as green bars; those fixations that have passed all our post hoc validity checks and have been stored as reliable fixations are marked as orange bars

The eyetracker records separate *x*- and *y*-coordinates for the right and left eyes. First, following Stampe (1993), these data were smoothed using a bilateral filtering algorithm written by Ed Vul (Frank, Vul, & Johnson, 2009; based on Durand & Dorsey, 2002). This algorithm eliminates jitter in fixation while preserving saccades (Frank et al., 2009). Because we found, on the basis of visual inspection, that samples for which only one eye was available were more likely to be inaccurate, these samples were excluded, and smoothing was performed only for those iterations in which *x-* and *y-* gaze coordinates were returned for both eyes. In Figs. 7 and 8, the top section shows the unsmoothed, raw data, and the section below shows the data after smoothing. During this process, data are converted into a single *x-* and *y-* position, averaged across both eyes.

### Interpolating

Interpolation was then performed to cover short segments of lost data. A duration of 150 ms was chosen as the longest period of missing data that was interpolated, in order to preclude the possibility of interpolating through a complete saccade–fixation–saccade sequence (the minimum time taken to program a saccade is 100–130 ms; Inhoff & Radach, 1998; Radach, Heller, & Inhoff, 1999). In Fig. 7, the letter “a” below the *x*-axis (at around 0.5 s) shows an instance where interpolation has been performed to cover a short segment of missing data. Also in Fig. 7, example “b” (at around 2.3 s) shows an instance of missing data that was too long to interpolate.

During interpolation, the average *x-* and *y*-coordinates were calculated since the start of the active fixation, and these positions were continued forward until the data came back online. At this point, the velocity was calculated between the last interpolated sample and the first sample after interpolation; if the velocity change was above our velocity threshold, it was judged that a saccade had taken place at some unknown point during the missing data. Since we cannot know when the saccade occurred during the missing period, in these cases we have therefore excluded the fixations before and after the missing segment.

### Velocity thresholding

The velocity (i.e., rate of change of reported POG) was then calculated independently for the rough and smooth gaze coordinates. In Figs. 7 and 8, the velocity based on the rough samples is drawn in black, and the velocity based on the smoothed samples is drawn in red. Increasing the velocity threshold decreases the proportion of “false positive” saccades that have to be filtered out at step 4 but increases the proportion of small saccades that are subthreshold and that fail to be detected (see the discussion in Holmqvist et al., 2011). We decided to set the velocity threshold at 35ºs^-1^, which is similar to that used by some researchers (e.g., Tatler & Vincent, 2008) but much lower than that used by others (e.g., Smeets & Hooge, 2003). In the first-pass processing, all sections where the velocity remained under this threshold were marked as possible fixations (marked as thick green lines on Figs. 7 and 8).

### Rejection of false fixations and saccades

Steps 1–3, discussed above, are standard in fixation detection algorithms. As was discussed, however, problems of unreliable eyetracker contact and poor quality data obtained from infants mean that a number of potentially false fixations are stored if only steps 1–3 are implemented. Therefore, we also implemented a number of post hoc validation criteria to check whether the fixations identified by our algorithms were genuine or artifactual. These post hoc verification techniques have not, to our knowledge, been implemented in any other fixation detection algorithms. The following checks were implemented.

#### Fixation is a complete fixation

Flickery or unreliable contact is pervasive in infant eyetracker data. Most commonly used fixation detection algorithms treat an instance in which eyetracker contact is lost during a fixation as signaling the end of that fixation. Thus, example “b” in Fig. 7 shows an instance where contact is lost for a period of time that is too large to interpolate across. Before and after this period of lost data, incomplete fixations have been stored (marked as green rectangles). Most commonly used algorithms would save these incomplete fixations along with the other fixations, even though their exact duration cannot be judged accurately on the basis of the data available. This leads to artificially short fixation durations and to the relationship between data quality and fixation duration that we illustrated in Figs. 3, 4, 5 and 6. In order to judge, therefore, whether a fixation was a complete one, we checked that saccades had been registered before and after that fixation without any intervening periods of lost data. Figure 8, example “f”, shows another example of two partial fixations (marked in green) that are not complete and have, therefore, not been stored.

The second problem was that of inconsistent or inaccurate reporting of POG, which we found can surpass the velocity threshold and lead to false positive saccades (see Figs. 5c and 6b). To identify these “false saccades,” we implemented an additional four criteria. This leads to some redundancy, since artifacts identified using one criterion are often also identified using other criteria. However, we decided that redundancy was not a problem in the present instance. The four criteria we implemented to identify false positive saccades were the following:

#### Displacement since the previous fixation is above threshold

One marker we noted for “false saccades” was that the location of the “fixation” after the “saccade” was the same as that before the “saccade.” Therefore, if two successive fixations had a Euclidean distance between them of less than 0.25º, they were both labeled as unreliable and were not stored (see example “c” in Fig. 8).

#### Average velocity during previous fixation is not above threshold

Another marker we noted for “false saccades” was that they often occurred during segments in which the reported POG appeared to be inaccurate over sustained periods. Therefore, at every saccade, the variance of the immediately preceding fixation was calculated; if above 12ºs^−1^, the saccade was considered to be from a segment of unreliable data, and the preceding and subsequent fixations were both labeled as unreliable and were not stored (see example “e” in Fig. 8).

#### Velocity in the three samples immediately preceding the saccade is not above threshold

In addition to calculating the average velocity for the whole preceding fixation, a moving window was also calculated of the three samples immediately preceding the saccade. If this was above a threshold (also set at 12ºs^−1^), the saccade was labeled as unreliable, and the fixations before and after were not stored (see also example “e” in Fig. 8).

#### Binocular disparity is not above threshold

An additional marker we noted as characteristic of “false” saccades was that the disparity between the positions reported by the left and right eyes was larger than normal. Example “c” in Fig. 8 shows an instance in which one iteration has returned a *y*-coordinate for one eye that is substantially (>3º) different from the *y*-coordinate for the iterations immediately before and after, while the *y*-coordinate for the other eye has remained the same. This is almost certainly artifactual—caused, for example, by the misidentification of a glint on the cornea arising from another light source in the room—yet even postsmoothing this has triggered our velocity-based saccade detection criterion. In order to avoid the mislabeling of saccades in this way, the binocular disparity (i.e., the difference between the POG reported for the left and right eyes) was calculated for the 60 ms immediately before the saccade. If the binocular disparity in any of the samples (either *x* or *y*) over this period was above a threshold (set with reference to our findings about the accuracy of the eyetracker at 3.6º), the saccade was labeled as unreliable, and fixations either side of that saccade were rejected.

#### Minimum temporal duration

The final post hoc criterion that we implemented was the elimination of fixations shorter than 100 ms. Although there is a considerable body of research within the adult literature that studies these very short, preprogrammed fixations (Inhoff & Radach, 1998; Nuthmann et al., 2010; Volkman, 1986; although this remains controversial, see Irwin, 1992; Manor & Gordon, 2003), we felt that studying fixations of <100 ms was beyond the limits of the accuracy of the eyetracker we were using. The threshold we used of 100 ms is higher than is sometimes used (e.g., Tatler & Vincent, 2008, 50 ms), although lower than that used by others (e.g., de Barbaro et al., 2011, 230 ms; Irwin, 1992, 150 ms; see Inhoff & Radach, 1998, for a discussion of this issue). As with all the thresholds described in this section, the user can change this setting in our algorithms if (s)he sees fit.

As a result of these post hoc verification checks, many of the segments marked as tentative fixations following velocity thresholding (marked as green bars on Figs. 7 and 8) were rejected as insufficiently trustworthy. Only those tentative fixations that passed all of our checks were stored as reliable fixations (marked as orange bars on Figs. 7 and 8). Thus, of the 10 fixations tentatively identified in Fig. 7, only 8 were retained as fixations. In Fig. 8 (which shows a segment of lower quality data) of the 14 segments tentatively identified as fixations, only 4 were deemed to be reliable by our criteria.

## Assessing the reliability of our analyses

**Fig. 9:**
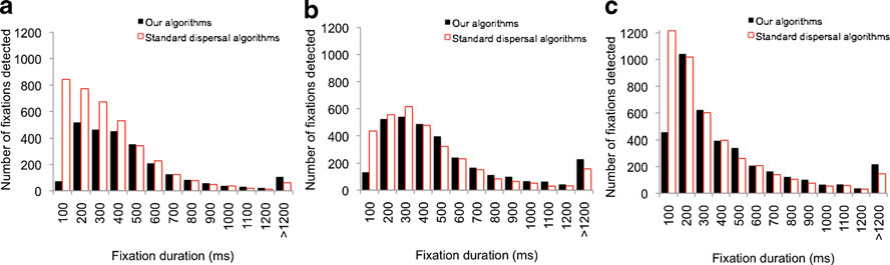
Frequency distributions for fixation durations obtained from three different age groups, comparing the results obtained from a standard dispersal algorithm with those obtained from our algorithms. **a** Six-month-olds. **b** Twelve-month-olds. **c**) Adults. The averages of the fixations obtained from each age group are shown in Table 1

**Fig. 10:**
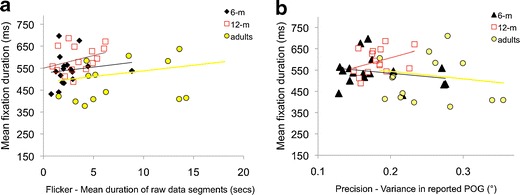
Quantifying how fixation duration varies as a function of data quality. The relationship between fixation durations as analyzed using our new algorithms and data quality, evaluated in two ways: **a** flicker, the mean duration of raw data segments, and **b** precision, the variance in reported point of gaze (POG). This recreates the analyses shown in Fig. 3

**Fig. 11:**
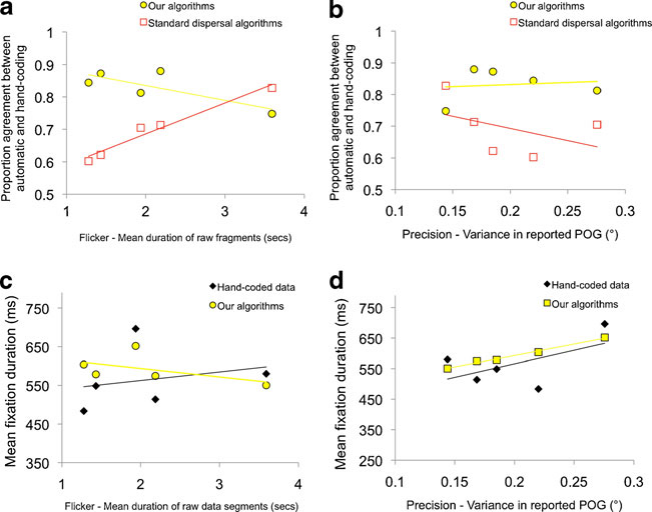
Quantifying performance of our algorithms relative to hand-coding as a function of data quality. Individual data-points show the results from individual participants whose data have been parsed using different processing techniques. **a** Relationship between flicker and proportion agreement between automatic and hand-coding. **b** Relationship between precision and proportion agreement between automatic and hand-coding. **c** Relationship between flicker and mean fixation duration as processed using both hand-coding and our algorithms. **d** Relationship between precision and mean fixation duration as processed using both hand-coding and our algorithms. This recreates the analyses shown in Fig. 4

**Fig. 12:**
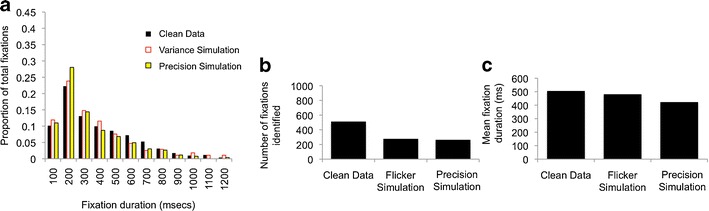
Evaluating the performance of our algorithm using simulations of the effect of flicker and precision. We reprocessed the same data shown in Fig. 5, but using our own algorithms rather than the standard dispersal algorithms. **a** Repeats the analysis shown in Fig. 5d). **b** Repeats the analysis shown in Fig. 5e. **c** Repeats the analysis shown in Fig. 5f

**Fig. 13:**
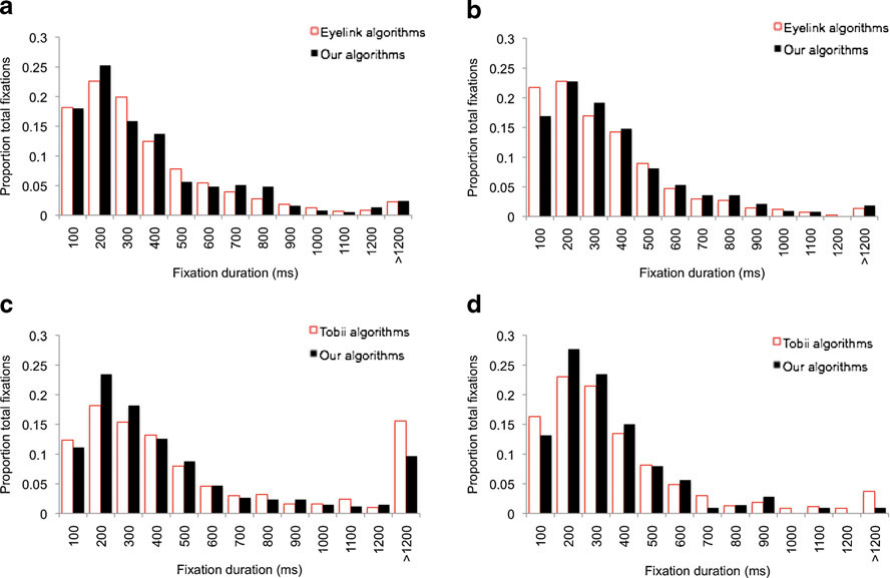
Frequency distributions of fixation durations of high-quality adult viewing data from two participants viewing the same stimuli on two eyetrackers. Viewing data have then been processed twice: first, using the manufacturer-supplied fixation detection algorithms, and second, using the new algorithms we present here. **a** Eyelink 1000, participant 1. **b** Eyelink 1000, participant 2. **c** Tobii TX300, participant 1. **d** Tobii TX300, participant 2

We evaluated the performance of our new fixation detection algorithms in a variety of ways. First, we compared the results of our new algorithms with those of standard dispersal algorithms on data from 6-month-old, 12-month-old, and adult viewers (Fig. 9 and Table 1). Second, we repeated the analyses presented in Figs. 3, 4, and 5, which look at the relationship between fixation duration as measured using our new algorithms and the quality of the raw data on which the analysis is conducted (Figs. 10, 11, and 12). Third, we repeated the “double processing” on a selection of higher quality adult data collected from two more modern eyetrackers—the Tobii TX300 and the Eyelink 1000—and compared the performance of our new algorithms with those of the manufacturer-supplied algorithms (Fig. 13 and Table 2).

**Table 1:** Averages of the fixation durations obtained using standard dispersal algorithms and using our new algorithms

		Mean (ms)	Mode (ms)	Median (ms)
6-month-olds	Clearview algorithms	400	100	339
	New algorithms	545	380	460
12-month-olds	Clearview algorithms	510	379	399
	New algorithms	604	380	480
Adults	Clearview algorithms	409	279	279
	New algorithms	521	280	380

**Table 2:** Averages of the fixation durations obtained using manufacturers’ algorithms and our new algorithms

		Mean (ms)	Mode (ms)	Median (ms)
Participant 1	Eyelink algorithms	393	201	336
	New algorithms	395	153	335
Participant 2	Eyelink algorithms	379	134	324
	New algorithms	401	194	345
Participant 1	Tobii algorithms	429	237	365
	New algorithms	413	263	343
Participant 2	Tobii algorithms	380	313	330
	New algorithms	378	270	320

The first analysis (Fig. 9 and Table 1) consists of reprocessing the viewing data that have already been analyzed in Fig. 3 using our new fixation detection algorithms. These data are from typically developing 6-month-old, 12-month-old, and adult viewers as they viewed a short battery of dynamic viewing material.

Figure 9 shows a comparison of the frequency distributions of the fixation durations obtained by the two parsing methods. Data from the three age groups have been presented in separate frequency distributions. The three figures show that consistently across the age groups, the standard dispersal algorithms report a greater total number of fixations than do our new algorithms and that these additional fixations appear to be almost exclusively very short fixations (mainly in the 100- to 200-ms range). There are also substantial differences in the mean, median, and mode of the distributions (see Table 1). The most egregious difference is in the 6-month-old group, for whom the quality of the raw data is lowest (see Fig. 3). In this age group, the standard dispersal algorithms have detected 3,990 fixations, whereas our algorithms have detected 2,829, a difference of 29%. There are substantial differences in the mean (400 vs. 545 ms), mode (100 vs. 380 ms), and median (339 vs. 460 ms) fixation durations returned by the two algorithms.

### Assessing the relationship between fixation duration as measured using our new algorithms and the quality of the raw data on which the analysis is conducted

Of the two sets of results presented in Fig. 9, which is closer to reality? In order to address this question, we repeated the analyses presented in Figs. 3, 4, and 5.

Figure 10 shows an analysis identical to that presented in Fig. 3. Across the three age groups, no significant relationships were observed between raw data segment duration and fixation duration as parsed using our new algorithms, although weaker, nonsignificant relationships do still remain [6months, *r*(1, 16) = .28, *p*(two-tailed) = .26; 12 months, *r*(1, 14) = .35, *p*(two-tailed) = .20; adults, *r*(1, 15) = .26, *p*(two-tailed) = .36]. Similarly, no significant relationships were observed between precision and fixation duration [6 months, *r*(1, 16) = −.23, *p*(two-tailed) = .38; 12 months, *r*(1, 14) = .37, *p*(two-tailed) = .17; adults, *r*(1, 14) = −.13, *p*(two-tailed) = .64]. Also notable from comparing Figs. 3 and 9 is that the range of mean fixation durations returned by our algorithms (360–710 ms) is much lower than that returned by the standard dispersal algorithms (160–770 ms).

### Comparing with hand-coded processing

Next, we repeated the analysis presented in Fig. 4, which looked at the reliability of automatic versus hand-processing as a function of data quality. Figure 11a shows how reliability (proportion of agreement between the hand and automatic data processing) varies as a function of data quality. Surprisingly, for the individual showing high data quality (mean raw fragment duration, 3.8), our new algorithms appear to be less reliable than the standard dispersal algorithms. Visual inspection of this individual’s data suggests that this is because this particular individual shows a high proportion of near-by but discrete fixations that are rejected as false negatives, due to our displacement criterion (see the Displacement Since the Previous Fixation Is Above Threshold section), but that are detectable by hand inspection. (There is nevertheless a close agreement on mean fixation duration between the hand- and the automatic coding for this individual, which suggests that those fixations being rejected as false negatives by our algorithm are not distorting the results.) Crucially, however, the results suggest that for lower quality data, the results of our new algorithms remain reliable (proportion of agreement, .8–.9); there appears to be no relationship between reliability of the algorithms and data quality, *r*(1, 4) = −.30, *p* = .62. This is in contrast to the results of the standard dispersal algorithms, whose reliability falls off rapidly in lower quality data. Figure 4b shows the relationship between precision and data quality as estimated using variance in reported POG. Again, the results suggest that for low-precision data (high variance in reported POG), the results of our new algorithms remain reliable (proportion of agreement, .8–.9), in contrast with the results of the standard dispersal algorithm, whose reliability is lower. Again, no relationship could be found between reliability of the algorithms and data quality, *r*(1, 4) = .13, *p* = .84, in contrast to the significant negative correlation reported in Fig. 4b.

Figure 4c and 4d shows a comparison of mean fixation durations as reported by hand-coding and our new algorithms. They show that some substantial differences do still exist between the results of our algorithms and those of the hand-coding, which may be attributable to the small volume of data that has been processed in this analysis. Crucially, however, the systematic interactions documented in Fig. 4c and 4d—that the standard dispersal algorithms tend to underestimate fixation durations for lower quality data and to overestimate fixation durations for higher quality data—appear absent for the results of our new algorithms.

### Comparing with simulation

Our final method for assessing the degree to which the performance of our new algorithms was affected by raw data quality was to repeat the same analysis as that presented in Fig. 5, which used data that had been distorted via a simulation to create flicker and low precision (Fig. 12).

In Fig. 5e, we noted that both manipulations had the effect of increasing the number of fixations detected by the standard dispersal algorithm, which appears to be caused by the algorithm identifying an increased number of incomplete, fragmentary fixations. Figure 12b shows that both manipulations have the opposite effect on the performance of our new algorithms: Instead of increasing the number of fixations identified, they reduce them. This is because of the post hoc validation checks incorporated into our new fixation detection algorithms, which lead to tentatively identified fixations being rejected if the post hoc validation checks indicate that they are insufficiently trustworthy (see Figs. 7 and 8 for examples). Decreasing the quality of the raw data has the effect, therefore, of decreasing the number of fixations that can confidently be identified. However, and in marked contrast to the analyses shown in Fig. 5d, f, the fixations being identified by our new algorithms are relatively more invariant to variations in data quality. The distribution of fixation durations identified is markedly similar between the cleaned and distorted data (Fig. 12a), in contrast to the performance of the standard dispersal algorithm (Fig. 5d). Mean fixation duration returned by our algorithms is 506 ms for the clean data, 480 ms for the flicker simulation, and 424 ms for the low-precision simulation (in contrast to 472 ms/240 ms/367 ms for the standard dispersal algorithms shown in Fig. 5). This suggests that, although far from perfect, the results of our new algorithms are relatively more robust to differences in raw data quality.

### Evaluating the performance of our algorithms on adult data

Hitherto, we have reported on the performance of our algorithms on data collected from a Tobii 1750 eyetracker, which is a widely used eyetracker for infant research. Next, we repeat the same comparison with two different eyetrackers, the 300Hz Tobii TX300 and the 1000 Hz Eyelink 1000, in order to see how well they perform on data that are of higher quality and of a higher temporal resolution. This analysis is conducted on adult data since the Eyelink 1000 (which requires the participant to rest their head on a chinrest) is not amenable to use with infants.

Viewing data were collected while a 600-s dynamic short film was viewed by two adult viewers. The material was presented on a TX300 eyetracker using Tobii Studio and on an Eyelink 1000 eyetracker using Experiment Builder. Both setups used a 21-in. screen at a viewing distance of 60 cm and a screen resolution of 1,024 × 768 pixels. Fixation parsing was performed using the fixation identification algorithms supplied by the eyetracker manufacturers; then raw viewing data were exported and reprocessed post hoc using our new algorithms (see Fig. 13 and Table 2). The Eyelink algorithm used the standard velocity, acceleration, and displacement thresholds of 30°/s, 8,000°/s^2^, and 0.1° (Eyelink User Manual, 2005). The TX300 data were parsed using Tobii Studio's standard fixation filter with a velocity threshold of 35 pixels per sample (equivalent to 64°/s at 300 Hz on a 800 × 600 pixel display subtending a viewing angle of 24°) and a distance threshold of 35 pixels (the algorithm interpolates across gaps <100 ms). First, calculations were conducted to examine data quality; the results suggested that for all measures, data quality from these adult viewers was high relative to the data presented in Fig. 3. For the Eyelink 1000, the proportion of unavailable data was less than .04 of the total viewing material presented for both participants, the mean duration of raw data fragments was 9,800 ms for participant 1 and 3,300 ms for participant 2, and average within-fixation variance was approximately 0.12° for both participants. For the TX300, the proportion of unavailable data was higher (*circa* .17) for both participants; the mean duration of raw data fragments was 2,100 ms for participant 1 and 1,500 ms for participant 2. Within-fixation variance was below 0.1° for both participants.

Figure 13 shows the results of the comparisons between fixation duration as parsed using the fixation detection algorithms provided by the eyetracker manufacturers and our new algorithms. As we predicted, given that the data we are analyzing are of high quality, all four comparisons are reasonably closely matched. The means and medians in particular show high reliability, with the largest difference between the results of our algorithms and those of the comparison algorithms being 22 ms (see Table 2). The modes are more sensitive and show lower test–retest reliability. The frequency distributions also show reasonably close matching of distributions. The comparison of the TX300 data (Fig. 13c, d) suggests that the manufacturer’s algorithms are reporting a higher proportion of very long (>1,200 ms) fixations, which may be due to the high velocity threshold (64°/s).

## Discussion

In this article, we examined fixation duration as parsed using the standard dispersal-based algorithms supplied by most eyetracker manufacturers and discussed possible links between this measure and interindividual differences in data quality. In order to examine this relationship, we quantified data quality in two ways: First, we assessed how flickery or unreliable the contact with the eyetracker was, and second. we assessed the precision—that is, the variance in the reported POG. Our investigations suggested that these parameters do not themselves correlate, suggesting that they represent separate dimensions of data quality. We also found that both dimensions of data quality differ substantially between infant and adult data (Fig. 3).

We presented results (Fig. 4) suggesting that fixation durations as parsed using the standard dispersal-based algorithms supplied by most eyetracker manufacturers are significantly influenced by both dimensions of data quality. Participants for whom contact with the eyetracker is more fragmentary or flickery are returned as showing significantly shorter fixation durations than participants for whom eyetracker contact was more reliable (Fig. 3a). And participants for whom the reporting of the POG was more imprecise also showed significantly shorter fixation durations (Fig. 3b).

We also discussed the reasons underlying these findings. In particular, we concentrated on two key problems that can lead to excess very short fixation durations being reported: first, storing fragmentary fixations as complete fixations (see Fig. 6a, Fig. 7 example “b” and Fig. 8 example “a”), and second, failing to identify instances in which the velocity threshold is surpassed not because of a genuine saccade, but instead because of inaccurate reporting of POG, leading to a displacement between one iteration and the next that surpasses the saccade threshold (see Figs. 6b and 8 example “e”).

We have presented new algorithms that we designed specifically to cope with these problems. These algorithms differ from previously published algorithms primarily in that they include a variety of post hoc verification checks that are conducted after identification of a fixation to identify and eliminate artifactual fixations from the data.

We have presented assessments of our algorithms in two sections. First, we assessed the performance of our algorithms on viewing data collected on a Tobii 1750 eyetracker while cohorts of 6-month-old, 12-month-old, and adult viewers viewed a battery of dynamic viewing material. Our analyses suggested that whereas systematic relationships exist between data quality and fixation durations as parsed using standard dispersal-based algorithms (Figs. 3, 4, and 5), no such relationships could be identified with the results of our algorithms (Figs. 9, 10, and 11). The results of the hand-coding of fixation durations appear to be closer for the performance of our new fixation detection algorithms than for those of the standard dispersal algorithms (Figs. 4 and 10).

We also assessed the performance of our algorithms on adult viewing data collected from an Eyelink 1000 and a Tobii TX300 eyetracker and compared the results of our algorithms with the results of the fixation identification filters supplied by the eyetracker manufacturers (Fig. 13). Our quantitative assessments of the quality of these data (degree of flicker and precision) suggested that it was of high quality, and we found that the results of our fixation-parsing algorithms matched closely with those of standard algorithms.

Some eye-tracking manufacturers are aware of the issues discussed in this article concerning simple dispersal or velocity-based algorithms and are attempting to incorporate more intelligent algorithms into the software provided with the eye-tracking systems. For example, Tobii Studio version 2.3 (and above; Tobii Studio 2.X User Manual, 2010) includes a modified I-VT (identification by velocity threshold) method that incorporates interpolation across gaps (75 ms by default), options for dealing with loss of one eye, noise reduction (median or mean of a three-sample moving window), velocity thresholds (30°/s over a 20-ms period), and the option to merge adjacent fixations (<0.5°) separated by a brief period of lost data (<75 ms) or to exclude short fixations (<60 ms). This should result in a significant improvement to the accuracy of fixations produced. However, the Tobii I-VT algorithm does not include the post hoc checks that we show are critical for dissociating fixation durations from data quality—for example, accepting only fixations begun and ended by saccades, or with minimum displacement from previous fixation, low velocity during fixation, and so forth.

A number of limitations remain to the algorithms and analyses we present here. First, the analyses we have presented in Figs. 9, 10, and 11 continue to show the same relationships between data quality and fixation duration as those identified in Figs. 3, 4, and 5 (albeit much weaker and nonsignificant). Visual inspection of the data (versions of which are available for download at http://www.cbcd.bbk.ac.uk/people/scientificstaff/sam) suggests that this is not because our fixation detection algorithms are incorrectly identifying fixations but, rather, because in low-quality data, there is less chance that long sections of clean data occurring, such as the long fixations that are frequent in infant viewing data, can reliably be identified.

Another limitation of the algorithms we present here is that they have no separate mechanism for identifying periods of smooth pursuit (cf., e.g., Berg, Boehnke, Marino, Munoz, & Itti, 2009; Nyström & Holmqvist, 2010). Within the limitations of the accuracy of the infant eyetrackers we are using, we could find no adequate means for identifying smooth pursuit; a solution that involved identifying periods during which the velocity was above 0 but lower than the saccade detection threshold was considered impractical due to the inconsistencies in reported POG discussed in this article.

## Plans for future work

The work we have presented here offers a method for studying the allocation of attention at the subsecond scale during unconstrained orienting, using naturalistic and seminaturalistic dynamic stimuli presented 2-D on a computer screen. A number of research questions can in the future be addressed using these techniques. For example, are there differences between infants and adults in the degree to which fixation durations are influenced by exogenous, stimulus-driven factors (such as the rate of change of low-level information at the point fixated) versus endogenous factors (such as the semantic content at the point fixated—e.g. face vs. nonface) (cf. Berg et al., 2009; Frank et al., 2009; Itti & Baldi, 2009; Mital, Smith, Hill, & Henderson, 2010)? And how do individual differences in attention control relate to the relative importance of endogenous versus exogenous factors in guiding gaze location (cf. de Barbaro et al., 2011; Frick, Colombo, & Saxon, 1999; Rose, Feldman, & Jankowski, 2011; Rose, Feldman, Jankowski, & Van Rossem, 2008, 2011; Wass et al., 2011)?

These techniques also offer a building block by which to pursue a future goal: to study the subsecond correlates of infants’ spontaneous orienting in “truly” naturalistic settings using a head-mounted eyetracker (Corbetta, Guan, & Williams, 2011; Franchak & Adolph, 2011; Franchak, Kretch, Soska, & Adolph, 2010; Smith, Yu, & Pereira, 2011). The ultimate aim of research in this field is to link attention as measured using nonnaturalistic, individual-trial experiments with attention in “the wild” (Smith et al., 2011). It is only once this goal has been achieved that the role that attention plays in mediating learning within both typical and atypical development can be fully understood. Longitudinal correlations have been documented, for example, between early attention control and subsequent language acquisition (Rose et al., 2008, 2009), but the possible causal pathways underlying these correlations remain largely unexplored. Do individual differences in attention control relate to altered spontaneous orienting in naturalistic social settings, and does this in turn relate to superior language acquisition? Similarly, in atypical development, early abnormalities have been noted using experimental settings in aspects of attention control within a number of disorder groups (e.g. Cornish, Cole, Longhi, Karmiloff-Smith, & Scerif, 2012a, 2012b; Cornish, Scerif, & Karmiloff-Smith, 2007; Elsabbagh et al., 2009; Holmboe et al., 2009; Scerif, Cornish, Wilding, Driver, & Karmiloff-Smith, 2005; van de Weijer-Bergsma, Wijnroks, & Jongmans, 2008), but it remains unknown whether (and if so, how) the laboratory-assessed differences relate to differences in spontaneous, “real-world” behaviors. It is only once accurate measures have been identified for studying attention in unconstrained, spontaneous settings that the true role that attention plays in mediated learning can be fully understood.
